# Impact of the COVID-19 pandemic on emergency hospital cancer admissions in a UK region

**DOI:** 10.1186/s12885-022-09932-3

**Published:** 2022-08-04

**Authors:** Helen Mitchell, Ben S. Alford, Simon O’Hare, Eamon O’Callaghan, Colin Fox, Anna T. Gavin

**Affiliations:** 1 Northern Ireland Cancer Registry, Centre for Public Health, Queen’s University Belfast, Belfast, Northern Ireland; 2 Centre for Public Health, Queen’s University Belfast, Belfast, Northern Ireland; 3 Macmillan Cancer Support, London, England

**Keywords:** Emergency admissions, cancer, COVID-19

## Abstract

**Background:**

The pandemic disrupted society and health services through lockdowns and resource reallocation to care for COVID-19 patients. Reductions in numbers of cancer patients having surgery, being diagnosed pathologically or via 2-week wait, and screening programs pauses have been described. The effect on emergency presentation, which represents an acute episode with poor outcomes, has not been investigated. This study explored the pandemic’s impact on emergency hospital admissions for cancer patients in a UK region.

**Methods:**

Hospital discharge data for cancer patients in Northern Ireland, which included route to admission, were analysed for the pandemic era in 2020 compared to averages for March to December 2017–2019, focusing on volume and route of emergency admissions by demography and tumour site.

**Findings:**

Compared with the pre-pandemic era, the number of cancer emergency admissions fell by 12·3% in 2020. Emergency admissions for cancer were significantly reduced when COVID-19 levels were highest (− 18·5% in April and − 16.8% in October). Females (− 15·8%), urban residents (− 13·2%), and age groups 0 to 49 and 65–74 years old (− 17%) experienced the largest decreases as did those with haematological (− 14·7%), brain and CNS (− 27·9%), and lung cancers(− 14·3%). Significant reductions in referrals from outpatient departments (− 51%) and primary care (− 43%) (*p* < 0·001**)** were counterbalanced by admissions from other routes including confirmed or suspected COVID-19 infection (increase 83·6%).

**Interpretation:**

Reductions in emergency admissions, and pathologically diagnosed cancers, as reported by the Northern Ireland Cancer Registry (NICR), indicate undiagnosed patients in the community which has implications for future workloads and survival. Data suggest undiagnosed cases may be higher for haematological, brain and CNS, and lung cancers and among females. Efforts should be made to encourage people with symptoms to present for diagnosis or reassurance.

**Funding:**

The NICR is funded by the Public Health Agency of Northern Ireland. This work was supported by Macmillan Cancer Support and uses data collected by health services as part of their care and support functions.

**Supplementary Information:**

The online version contains supplementary material available at 10.1186/s12885-022-09932-3.

## Background

On 23rd March 2020, in response to a novel coronavirus (SARS-CoV-2), causing a disease known as COVID-19, national lockdowns were implemented in many countries including the United Kingdom (UK), where the overarching message was “Stay Home, Protect the NHS, Save Lives” [1]. Many health care providers diverted resources towards the treatment and care of COVID-19 patients. Furthermore, national cancer screening programmes postponed routine invitations to reduce infection risk while primary care offered virtual triage, limiting direct access to buildings and in person consultations [2–4]. Reductions in the numbers of cancer patients having surgery, being diagnosed pathologically or via 2-week wait, have been described [5]. It would be expected that these changes would lead to increased presentation via Accident and Emergency. Before the pandemic, emergency presentation was the route to cancer diagnosis for about 1 in 5 cancer cases in Northern Ireland (NI) (more so for older persons, patients in socially deprived areas, colon, and lung cancer patients) and poorer net survival rates for patients diagnosed via emergency routes were highlighted [6]. This aligned with earlier work in England [7] which showed that across all cancer types, 1-year relative survival was significantly lower for Emergency Presentation cases compared to other routes.

### Research in context

#### Evidence before this study

Previous evidence indicated that the first wave of the pandemic had a profound impact on the cancer services, with UK data reporting an 82% reduction in screening services, a 70% reduction on two-week wait system, and a 40% reduction in numbers of cancer patients receiving surgery [5, 8].

#### Added value of this study

This first study on the impact of COVID-19 on emergency cancer admissions indicates that in addition to fall off in cases presenting via screening there was also a reduction in presentations via emergency departments.

#### Implications of all the available evidence

These figures indicate a further deficit of cancer patients presenting for diagnosis and care. Additional resource will be required to investigate treat and provide holistic personalised support to the backlog of patients. A sustained campaign to increase symptom awareness and presentation to primary care is required.

### Data collection and management

Data were obtained for cancer related emergency admissions between March to December 2017–2019 (‘Pre-COVID’ era) and March to December 2020 (‘During COVID’ era) from the Patient Administration System (PAS); datasets provided to the N. Ireland Cancer Registry (NICR) by Health Trusts as part of the routine cancer registration processes.

These anonymised PAS data provided details of patients’ International Classification of Diseases (ICD) code, their demographics including age group: (‘0 to 49’, ‘50 to 64′, 65 to 74′, and ‘75+’) and sex, with deprivation quintile (1 = least deprived - 5 = most deprived) and rurality based on postcode of residence [9] before anonymisation.

As these were raw data that had not been through the normal routine checks that cancer registrations require, we have a mix of incident and prevalent cases. We grouped all cancer related emergency admissions into four route categories: ‘Accident and Emergency’, ‘General Practitioner’, ‘Other Emergencies’ (including critical care units, home visits, other and COVID-19 admissions), and ‘Outpatient Department’. Tumour Site was determined by ICD 10 code [10] allocated on hospital discharge or death.

### Inclusion and exclusion criteria

**Fig. 1:**
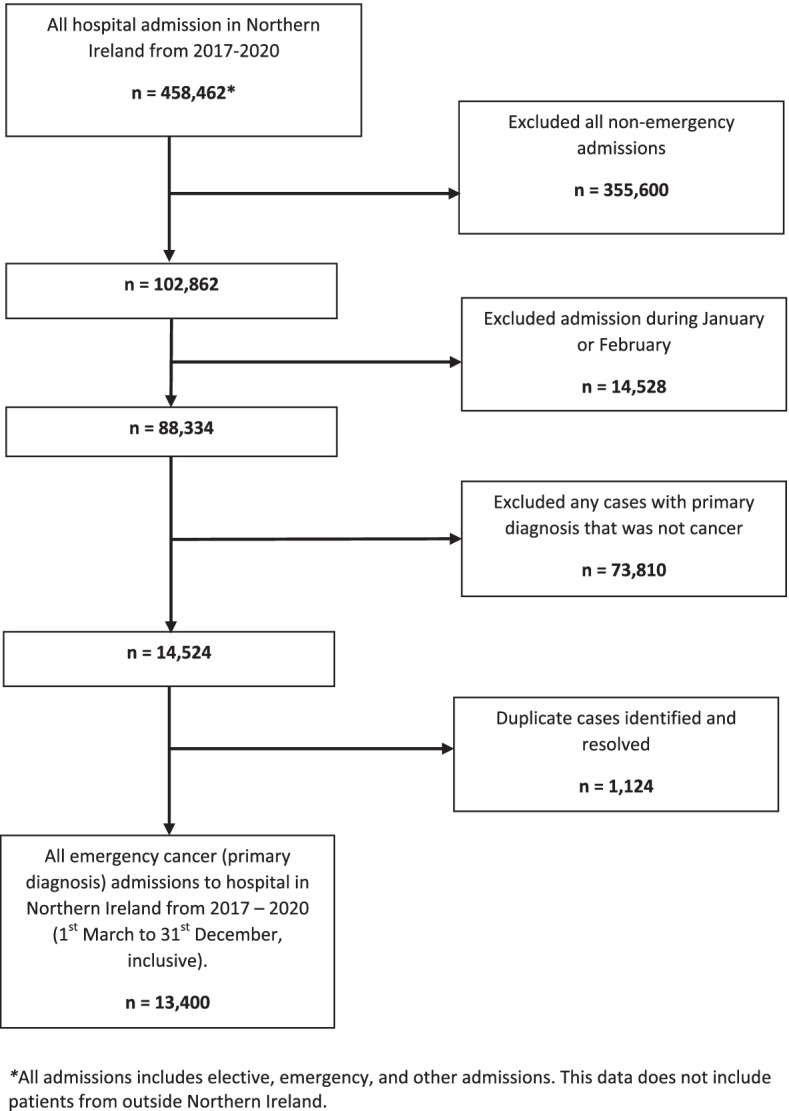
Flow diagram outlining how many cases were excluded from the study following the exclusion criteria

The same criteria were applied to both the ‘Pre-COVID’ era and the ‘During COVID’ patient cohorts. Figure 1 outlines the number of cases removed due to exclusion criteria, leaving 13,400 cases over the 4 years that met our criteria (2017 = 3432, 2018 = 3471, 2019 = 3466, and 2020 = 3031).

## Data analysis

Data analysis was completed using the Stata Software (Stata Corp LLC. TX. Software Release: 17). Binomial probability tests (with 95% confidence intervals) were used to analyse month of admission and tumour site.

This allowed us to investigate if the ‘During COVID’ patient cohort admissions represented the expected 25% of admissions over the four-year period studied. If there was no change in emergency admissions during the first year of the pandemic, we would expect each of the 4 years under analysis to represent around 25% of the total number of emergency admissions for the whole period.

Other categorical variables were analysed using Pearson’s chi-squared test and if significant, adjusted residuals were used to understand from where the associations originated, and Cramer’s V was used to show the strength of association [11].

### Ethical approval

This study was conducted on anonymised data in accordance with NICR Confidentiality and Data Protection Policies. The NICR has ethical approval from the Office for Research Ethics Committees of Northern Ireland (Ref: 12/NI/0132).

## Results

Compared to the average preceding 3 years, in 2020 the number of cancer related hospital admissions from any route fell by 14.5%. There were on average 3456 emergency cancer admissions per year across the ‘Pre-COVID’ cohort, and 3031 admissions ‘During COVID’: a decrease of 12·3%, representing a fall from 183·4 to 159·4 admissions per 100,000 people [13].

### Month of admission

Monthly emergency cancer admissions were significantly reduced when COVID-19 levels were highest; at the start of the pandemic (i.e., − 15·6% in March 2020) and in December 2020 when admissions dropped to the nadir (− 33·9%) - see Table 1), when the number of daily confirmed cases surged to over 2000 per day, and a third lockdown was initiated.

**Table 1:** Number of emergency admissions by month of admission between the pre-COVID (2017–2019) and during COVID (2020) cohorts, with the results of binomial probability tests

Month of Emergency Admission	Patient Cohorts		Percentage change in Admissions from Pre-COVID to During COVID	Binomial Probability Test Results are 2020 levels significantly different from the expected 25%? (95% Confidence Intervals)
	Pre-COVID (2017–2019 Average)	During COVID (2020)		
March	353 (10·2%)	298 (9·8%)	- 15·6%	***p =*** **0·005**, 22·0% (19·8% to 24·3%)
April	351 (10·2%)	286 (9·4%)	- 18·5%	***p =*** **0·001**, 21·4% (19·2% to 23·7%)
May	354 (10·2%)	319 (10·5%)	- 9·9%	*p =* 0·056, 23·1% (20·9% to 25·4%)
June	350 (10·1%)	351 (11·6%)	+ 0·3%	*p =* 0·539, 25·1% (22·8% to 27·4%)
July	376 (10·9%)	360 (11·9%)	- 4·3%	*p =* 0·246, 24·2% (22·0% to 26·5%)
August	359 (10·4%)	335 (11·1%)	- 6·7%	*p =* 0·138, 23·7% (21·5% to 26·0%)
September	336 (9·7%)	311 (10·3%)	- 7·4%	*p =* 0·119, 23·6% (21·3% to 26·0%)
October	340 (9·8%)	283 (9·3%)	- 16·8%	***p =*** **0·003**, 21·7% (19·5% to 24·1%)
November	340 (9·8%)	291 (9·6%)	- 14·4%	***p =*** **0·009**, 22·2% (20·0% to 24·5%)
December	298 (8·6%)	197 (6·5%)	- 33·9%	***p <*** **0·001**, 18·1% (15·8% to 20·5%)
**Total**	**3457 (100%)**	**3031 (100%)**	**- 12·3%**	

### Route to emergency admission

Despite an overall reduction of 12·3%, reductions in the source of emergency admission was mainly from primary care (-42·7%), outpatient departments (-51%) and the A&E department (-15·2%). This while emergency admissions for other sources (Critical Care Units, Home Visits and suspected or confirmed COVID-19 infection) increased by 83·6% (see Table 2).

**Table 2:** Proportion of emergency admissions by route to emergency admission for pre-COVID (2017–2019) and during COVID (2020) patient cohorts

Route to Emergency cancer related Admission	Patient Cohorts		Percentage Change in Admissions from Pre-COVID to During COVID	Pearson’s Chi-squared Result
	Pre-COVID (2017–2019 Average)	During COVID (2020)		
Accident and Emergency (Includes walk-ins, ambulance admissions, etc.)	2657 (76·9%)	2252 (74·3%)	- 15·2%	*Χ*^*2*^ (3, *N* = 6487) = 158·26. ***p <*** **0·001**. Cramer’s V = 0·156.
General Practitioner	131 (3·8%)	75 (2·5%)	- 42·7%	
Other Emergencies (COVID-19, Critical Care Units, Home Visits, Other)	280 (8·1%)	514 (17·0%)	+ 83·6%	
Outpatient Department	388 (11·2%)	190 (6·3%)	- 51·0%	
**Total**	**3456 (100%)**	**3031 (100%)**	**- 12·3%**	

### Tumour site

There were significant reductions in emergency admissions for most tumour sites, especially brain (-27·9%), head and neck (-27.4%), female breast (-26·4%), male (-20·8%) and female (-25.0%) genitalia, and lung (-14·7%). There were non-significant changes observed in the number of emergency admissions for liver (increase of 8%), and no change for pancreas and upper GI cancers.

### Socioeconomic status

There were declines in the total admissions in each deprivation quintile (as depicted in Supplementary material, Table 3). Pre COVID-19, the proportions of cancer admissions via A&E was similar in each of the 5 socioeconomic groups (SEG) at 20%, while during the COVID-19 era the proportions were significantly lower among the higher SEG (17·6%) and higher among the lower SEG (22·1%) (*p* = 0·002).

**Table 3:** Number of emergency admissions by tumour site between the pre-COVID (2017–2019) and during COVID (2020) cohorts

Tumour Site and ICD10 code	Patient Cohorts		Percentage Change in Admissions from Pre-COVID to During COVID	Binomial Probability Test Results (95% Confidence Intervals)
	Pre-COVID (2017–2019 Average)	During COVID (2020)		
Brain and CNS (C70–72, C75·1–75·3, D32–33, D35·2–35·4, D42–43, D44·3–44·5)	204 (5·9%)	147 (4·9%)	- 27·9%	***p <*** **0·001**, 19·2% (16·5% to 22·2%)
Breast (C50)	72 (2·1%)	53 (1·7%)	- 26·4%	***p =*** **0·024**, 19·7% (15·1% to 25·0%)
Colorectal (C18–20)	331 (9·6%)	285 (9·4%)	- 13·9%	***p =*** **0·013**, 22·3% (20·0% to 24·7%)
Female Genitalia (C53–56)	108 (3·1%)	81 (2·7%)	- 25·0%	***p =*** **0·011**, 20·0% (16·2% to 24·3%)
Haematological (C81–86, C90–95)	505 (14·6%)	431 (14·2%)	- 14·7%	***p =*** **0·001**, 22·1% (20·3% to 24·1%)
Head and Neck (C00–14, C30–32)	106 (3·1%)	77 (2·5%)	- 27·4%	***p =*** **0·005**, 19·5% (15·7% to 23·8%)
Liver (C22)	99 (2·9%)	107 (3·5%)	+ 8·1%	*p =* 0·782, 26·6% (22·3% to 31·2%)
Lung (C33–34)	540 (15·6%)	463 (15·3%)	- 14·3%	***p =*** **0·001**, 22·2% (20·5% to 24·1%)
Male Genitalia (C61, C63)	101 (2·9%)	80 (2·6%)	- 20·8%	***p =*** **0·036**, 20·9% (17·0% to 25·4%)
Other (all other ‘C’ codes)	206 (6·0%)	193 (6·4%)	- 6·3%	*p =* 0·221, 23·8% (20·9% to 26·9%)
Pancreas (C25)	150 (4·3%)	150 (5·0%)	0·0%	*p =* 0·513, 25·0% (21·6% to 28·6%)
Skin (C43–44)	27 (0·8%)	25 (0·8%)	- 7·4%	*p =* 0·397, 23·4% (15·7% to 32·5%)
Unknown Primary (C77–80)	677 (19·6%)	635 (21·0%)	- 6·2%	*p =* 0·081, 23·8% (22·2% to 25·5%)
Upper GI Tract(C15–16)	202 (5·9%)	204 (6·7%)	+ 1·0%	*p* = 0·567, 25·2% (22·3% to 28·3%)
Urinary Tract (C64–68)	124 (3·6%)	98 (3·2%)	- 21·0%	***p =*** **0·019**, 20·8% (17·2% to 24·8%)
**Total**	**3452 (100%)**	**3029 (100%)**	**- 12·3%**	

### Rurality

There were no significant differences in the reduction in admissions rates between patients from rural (-11·3%) and urban areas (-13·2%).

### Gender and age

The total number of emergency admissions in 2020, compared to pre-Covid rates, fell by 9·2% for males and 15·8% for females. The largest reductions were in those aged 0–49 (-16 ·9%) and 65–74 (-16·5%) and the smallest in those aged 50–64 (-12·8%) and aged 75 (-7·5%).

**Fig. 2:**
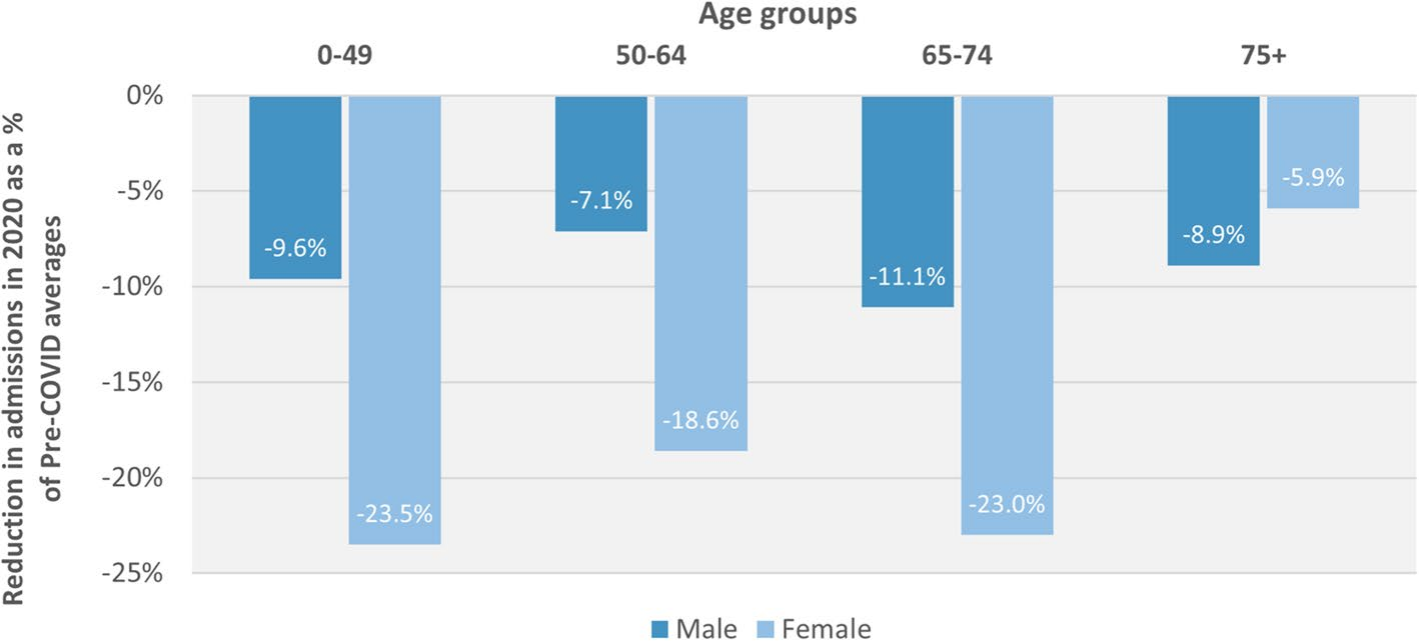
Reductions in emergency admission rates from Pre-COVID to During COVID period, by age and gender

Females had larger reductions than males for all age groups, except for the 75+ category, as summarised in Fig. 2.

## Interpretation

Emergency admission represents a failure of cancer prevention, early diagnosis or care of existing cancer patients and is associated with late stage at diagnosis and poorer survival.

We have demonstrated a reduction in emergency admission of all and cancer patients during the COVID-19 pandemic, with patterns related to periods of lockdown, stay at home messages and cessation of screening services. These main events coupled with patients’ fear of contracting COVID-19 when visiting hospital are thought to have had a profound impact on patient’s ability to receive timely cancer care. When compared to the same time period in 2019, it is estimated that there was a decrease of 50% in urgent cancer referrals across NI during the peak of the first wave, which is the equivalent of approximately 7500 fewer referrals during that time span [14]. Furthermore, between March and July 2020, there were approximately 900 fewer pathology samples indicating cancer [15] and a 52% reduction in patients commencing their cancer treatments across NI. The reductions in pathologically diagnosed cancers has been sustained and impacted by further increases in COVID cases.

A reduction in emergency admissions during normal working would be welcome, as survival from emergency admissions is poor compared with other routes to diagnosis [7], but this reduction indicates a potential loss of patients who require diagnosis and care. Unfortunately, the biggest decrease was in those entering the system from primary care and outpatient departments, reflecting changes in the availability and access to these services where early warning symptoms are most likely to be detected.

The 12·3% reduction in total emergency admissions documented in this study is in line with that reported during the early phase of the pandemic in England with a 12% decline in cancer patients seeking GP appointments in 2020 [5] and reductions in outpatient attendances [16]. It is likely that there has been a similar undocumented reduction in cancer emergency admissions throughout the UK.

The impact of COVID on emergency admissions is also evident with patients more likely to be admitted via ‘other emergency routes’ such as Critical Care Units, Home Visits and suspected/confirmed COVID-19 infection in 2020, than in previous years [17].

Comparisons by tumour site revealed that there were significant decreases in the number of patients presenting as an emergency for brain and CNS, breast, colorectal, female genitalia, haematological, head and neck, lung, male genitalia, and urinary tract cancers. These likely reflect a reduction in the availability of specific health care services throughout lockdown [18], patients not seeking help due to feeling they would be a burden on the healthcare system [5], and the patients’ fear of COVID-19 [19]. A recent paper looking at global impact of COVID-19 on colorectal cancer care found that suspension of multidisciplinary team meetings, staff absence, reallocation of staff and resources to COVID-19 care and lack of personal protective equipment significantly impacted the diagnosis and treatment of colorectal cancer [20].

Additionally, patients were advised not to attend A&E or their GP if they had COVID-19 symptoms [12]. Several cancer symptoms are very similar to those for COVID-19 for example; a new continuous cough or breathlessness for lung cancer [21]; fever, chills, and fatigue for haematological cancers [22]; a loss of smell for nose and sinus cancer, and shortness of breath for laryngeal cancer [23], so the patient or health service may have mistakenly believed presenting patients had the effects of COVID-19. There was no change in the number of emergency admissions of pancreatic cancers. This is likely to be due to very few patients being symptomatic, or having indiscernible symptoms in the early stages of the disease, thus patients with pancreatic cancer often only receive a diagnosis when the cancer is advanced and emergency medical attention is required [24].

One explanation for the large decrease in admissions in the least deprived SEG could be the increased likelihood they have access to private healthcare options, which may have reduced the need to present as an emergency case to hospital. The most deprived SEG are less likely to have this as an option and access to both face-to-face and telehealth GP appointments was difficult during lockdown [25] and thus could result in symptoms progressing to the point of an emergency [26]. Previous research into the routes to diagnosis in NI [6] indicated that in the least deprived SEG 17% were diagnosed as a result of an A&E visit and this increased to 23% in the most deprived SEG, therefore it may be reasonable to assume that there will be an increase in the number of cancers diagnosed via A&E visits in the most deprived SEG during the pandemic.

Females were more heavily impacted by the pandemic with a greater reduction in the number of admissions (-15·8% compared to − 9·2% for males). The data examined included new and prevalent cancer admissions and may reflect the higher prevalence of female than male cancer patients [27]. It is unlikely that the reduction in screening services impacted on emergency presentations, as screening usually picks up early disease when in early stages, however it may reflect the volume of reductions in breast and gynaecological cancers and/or behaviour related to attitudes towards receiving healthcare. A recent study in England [28] found that males were significantly more likely to have an ‘avoidable’ admission compared to females. This could potentially explain why fewer females presented as an emergency in 2020, as they felt like they would be a burden on the NHS [5]. The age group that showed the smallest decrease was the ‘75+’, which is perhaps because the older generation are more vulnerable to COVID-19 and therefore are probably more reluctant to seek medical treatment unless urgent [29]. This could mean that they would ignore or be less likely to notice milder symptoms, leading to a more severe stage of cancer that would require emergency medical attention.

The major strength of the current study is the use of data from a nation-wide population-based registry. It also separated admissions which may have been linked with a diagnosis of COVID-19 in the ‘other’ group. However, it reflects only A&E attendances which resulted in a hospital admission and does not count patients who would have attended A&E but not required admission. It contains a mix of incident and prevalent cases and further study is planned to determine the impact on new and existing patients and over time the impact on survival.

This disruption affected patients receiving cancer care and is predicted to have long term effects as early detection and treatment is important for survival. Additionally, evidence suggests [30] that having a COVID-19 infection also impacts on survival outcomes for cancer patients, either due to undiagnosed cancer caused by symptom overlap with COVID-19, or increased severity of COVID-19 infection due to the diminished immune system of cancer patients undergoing treatment, potentially increasing their risk of poorer survival outcomes.

In conclusion, we have documented a deficit of cancer patients. Meeting the needs of these patients, and cancer patients whose treatment was disrupted by the pandemic, will not be easily achieved. The Minister for Health in Northern Ireland outlined that whilst Covid-19 was still present it was ‘no easy task to build services back up to pre-existing levels’ and ‘this will impact heavily on our capacity in the system to provide appointments, diagnostic tests, operations, and a wide range of other services’ [31].

In order to recover and identify persons in whom a cancer diagnosis has been missed there will need to be ongoing provision of additional resources to protect cancer services from the impact of further disruptions from surges in COVID-19 infections, achieve the capacity needed to keep Covid-protected services running, to increase capacity and reduce the cancer service backlog, and ensure the ongoing and timely publication of data showing the scale of the backlog caused at local and national level as well as progress in recovering services, as recommended by Macmillan [14].

It is also critical that people with concerns about suspected cancer symptoms, or worsening symptoms for existing patients, make an appointment with their GP. This will require a sustained campaign to encourage symptom awareness especially for those at highest risk (e.g., lung cancer).

## Supplementary Information

**Additional file 1. Supplementary Material** **Table 1.** A summary of all emergency admissions cases included in the study by year of admission for months March to December only, sex, age, and tumour site. NB: In-situ tumours have been moved to ‘Other’ in this table as the reported values were less than five. **Supplementary Material Table 2.** The Pearson’s chi-squared result’s adjusted residuals and Cramer’s V for route to emergency admission by patient cohort. **Supplementary Material Table 3.** The Pearson’s chi-squared result’s adjusted residuals and Cramer’s V for deprivation quintile by patient cohort*.* **Supplementary Material Table 4**. Changes in admission rates between the two cohorts by rurality. **Supplementary Material Table 5.** Changes in admission rates between the two cohorts, by age and gender.

## Data Availability

The datasets generated and/or analysed during the current study are not publicly available due the limits of the ethical approval granted to the NICR to share patient level data. Anonymised, non-patient level data can be made available from the corresponding author on reasonable request.

## Abbreviations

PAS: Patient Administration System
NI: Northern Ireland
NICR: N. Ireland Cancer Registry
ICD 10: International Classification of Diseases version 10
GI: Gastro-intestinal
CNS: Central Nervous System
SEG: Socioeconomic group
A&E: Accident and Emergency
GP: General Practitioner
