# Supplementary material for: Impact of the COVID-19 pandemic on emergency hospital cancer admissions in a UK region
Source: BMC Cancer. 2022 Aug 4;22:850. doi: 10.1186/s12885-022-09932-3 (PMC9351130; doi:10.1186/s12885-022-09932-3)
Supplement: Supplementary file 1 — Additional file 1. Supplementary Material Table 1. A summary of all emergency admissions cases included in the study by year of admission for months March to December only, sex, age, and tumour site. NB: In-situ tumours have been moved to ‘Other’ in this table as the reported values were less than five. Supplementary Material Table 2. The Pearson’s chi-squared result’s adjusted residuals and Cramer’s V for route to emergency admission by patient cohort. Supplementary Material Table 3. The Pearson’s chi-squared result’s adjusted residuals and Cramer’s V for deprivation quintile by patient cohort. Supplementary Material Table 4. Changes in admission rates between the two cohorts by rurality. Supplementary Material Table 5. Changes in admission rates between the two cohorts, by age and gender. [file 12885_2022_9932_MOESM1_ESM.docx]

**Supplementary Material**

**Supplementary Material, Table 1:** A summary of all emergency admissions cases included in the study by year of admission for months March to December only, sex, age, and tumour site. NB: In-situ tumours have been moved to ‘Other’ in this table as the reported values were less than five.

|  |  | **2017** | **2018** | **2019** | **2020** | **Total** |
| --- | --- | --- | --- | --- | --- | --- |
| **Sex** | Male | 1,795 (52·3%) | 1,856 (53·5%) | 1,819 (52·5%) | 1,655 (54·6%)  -9·2% | **7,125 (53·2%)** |
|  | Female | 1,632 (47·6%) | 1,611 (46·4%) | 1,643 (47·4%) | 1,372 (45·3%)  -15·7% | **6,258 (46·7%)** |
|  | Unknown | 5 (0·1%) | 4 (0·1%) | 4 (0·1%) | 4 (0·1%) | **17 (0·1%)** |
|  |  |  |  |  |  |  |
| **Age** | 0 to 49 | 361 (10·5%) | 380 (10·9%) | 322 (9·3%) | 294 (9·7%)  -17% | **1,357 (10·1%)** |
|  | 50 to 64 | 834 (24·3%) | 882 (25·4%) | 827 (23·9%) | 739 (24·4%)  -12·8% | **3,282 (24·5%)** |
|  | 65 to 74 | 979 (28·5%) | 944 (27·2%) | 1,001 (28·9%) | 813 (26·8%)  -16·58% | **3,737 (27·9%)** |
|  | 75 + | 1,253 (36·5%) | 1,264 (36·4%) | 1,316 (38·0%) | 1,181 (39·0%)  -7·56% | **5,014 (37·4%)** |
|  | Unknown | 5 (0·1%) | 1 (<0·1%) | 0 (0·0%) | 4 (0·1%) | **10 (<0·1%)** |
|  |  |  |  |  |  |  |
| **Tumour Site** | Brain and CNS (C70-72, C75·1-75·3, D32-33, 35·2-35·4, D42-43, D44·3-44·5) | 207 (6·0%) | 196 (5·6%) | 214 (6·2%) | 147 (4·8%)  -28·5% | **764 (5·7%)** |
|  | Breast (C50) | 81 (2·4%) | 70 (2·0%) | 65 (1·9%) | 53 (1·7%)  -26·4% | **269 (2·0%)** |
|  | Colorectal (C18-20) | 299 (8·7%) | 347 (10·0%) | 347 (10·0%) | 285 (9·4%)  -13·9% | **1,278 (9·5%)** |
|  | Female Genitalia (C53-56) | 107 (3·1%) | 112 (3·2%) | 104 (3·0%) | 81 (2·7%)  -24·4% | **404 (3·0%)** |
|  | Haematological (C81-86, C90-95) | 464 (13·5%) | 509 (14·7%) | 542 (15·6%) | 431 (14·2%)  -14·7% | **1,946 (14·5%)** |
|  | Head and Neck (C00-14, C30-32) | 119 (3·5%) | 101 (2·9%) | 98 (2·8%) | 77 (2·5%)  -27·4% | **395 (2·9%)** |
|  | Liver (C22) | 96 (2·8%) | 99 (2·9%) | 101 (2·9%) | 107 (3·5%)  -8·4% | **403 (0·3%)** |
|  | Lung (C33-34) | 553 (16·1%) | 517 (14·9%) | 550 (15·9%) | 463 (15·3%)  -14·3% | **2,083 (15·5%)** |
|  | Male Genitalia (C61, C63) | 100 (2·9%) | 99 (2·9%) | 103 (3·0%) | 80 (2·6%)  -20·5% | **382 (2·9%)** |
|  | Other (*All other* 'C' *codes,* D05-06*)* | 236 (6·9%) | 189 (5·4%) | 200 (5·8%) | 195 (6·4%)  -6·4% | **820 (6·1%)** |
|  | Pancreas (C25) | 179 (5·2%) | 140 (4·0%) | 132 (3·8%) | 150 (4·9%)  -0·2% | **601 (4·5%)** |
|  | Skin (C43-44) | 32 (0·9%) | 30 (0·9%) | 20 (0·6%) | 25 (0·8%)  -8·5% | **107 (0·8%)** |
|  | Unknown Primary (C77-80) | 637 (18·6%) | 730 (21·0%) | 665 (19·2%) | 635 (21·0%)  -6·3% | **2,667 (19·9%)** |
|  | Upper GI Tract (C15-16) | 211 (6·1%) | 199 (5·7%) | 196 (5·7%) | 204 (6·7%)  1·0% | **810 (6·0%)** |
|  | Urinary Tract (C64-68) | 111 (3·2%) | 133 (3·8%) | 129 (3·7%) | 98 (3·2%)  -21·2% | **471 (3·5%)** |
| **Total** | | **3,432 (25·6%)** | **3,471 (25·9%)** | **3,466 (25·9%)** | **3,031 (22·6%)**  **-12·3%** | **13,400 (100%)** |

**Supplementary Material, Table 2:** The Pearson’s chi-squared result’s adjusted residuals and Cramer’s V for route to emergency admission by patient cohort.

| **Method of Admission** | **Detail** | **Patient Cohorts** | |
| --- | --- | --- | --- |
|  |  | **Pre-COVID**  **(2017 - 2019 Average)** | **During COVID**  **(2020)** |
| Accident and Emergency | % within Patient Cohort | 76·9 % | 74·3 % |
|  | Adjusted Residual | **2·4** | **- 2·4** |
| General Practitioner | % within Patient Cohort | 3·8 % | 2·5 % |
|  | Adjusted Residual | **3·0** | **- 3·0** |
| Other Emergencies  (COVID-19, Critical Care Units, Home Visits, Other) | % within Patient Cohort | 8·1 % | 17·0 % |
|  | Adjusted Residual | **- 10·9** | **10·9** |
| Outpatient Department | % within Patient Cohort | 11·2 % | 6·3 % |
|  | Adjusted Residual | **7·0** | **- 7·0** |
| **Symmetric Measure** | | ***Cramer’s V* = 0·156.**  ***p*<0·001.** | |

**Supplementary Material, Table 3:** The Pearson’s chi-squared result’s adjusted residuals and Cramer’s V for deprivation quintile by patient cohort*.*

| **Deprivation Quintile** | **Detail** | **Patient Cohorts** | |
| --- | --- | --- | --- |
|  |  | **Pre-COVID**  **(2017 - 2019 Average)** | **During COVID**  **(2020)** |
| Quintile 1 (Least Deprived) | % within Patient Cohort | 19·1 % | 17·6 % |
|  | Adjusted Residual | 1·5 | - 1·5 |
| Quintile 2 | % within Patient Cohort | 20·0 % | 17·4 % |
|  | Adjusted Residual | **2·7** | **- 2·7** |
| Quintile 3 | % within Patient Cohort | 19·9 % | 22·6 % |
|  | Adjusted Residual | **- 2·6** | **2·6** |
| Quintile 4 | % within Patient Cohort | 21·0 % | 20·4 % |
|  | Adjusted Residual | 0·6 | - 0·6 |
| Quintile 5 (Most Deprived) | % within Patient Cohort | 20·0 % | 22·1 % |
|  | Adjusted Residual | **- 2·1** | **2·1** |
| **Symmetric Measure** | | **Cramer’s V = 0·051.**  ***p*=0·002.** | |

**Supplementary Material, Table 4:** Changes in admission rates between the two cohorts by rurality

| **Variables** | | **Patient Cohorts** | | **Percentage change in Admissions from Pre-COVID to During COVID** | **Pearson’s**  **Chi-squared Results** |
| --- | --- | --- | --- | --- | --- |
|  |  | **Pre-COVID**  **(2017 – 2019 Average)** | **During COVID**  **(2020)** |  |  |
| **Rurality** | Rural | 1,154 (33·4 %) | 1,024 (33·9 %) | - 11·3 % | *Χ^2^* (1, N = 6,470) = 0·18.  *p*=0·671. |
|  | Urban | 2,298 (66·6 %) | 1,994 (66·1 %) | - 13·2 % |  |
|  | **Total** | **3,452 (100 %)** | **3,018 (100 %)** | **- 12·6 %** |  |

| **Variables**  **(Gender and Age groups)** | | | **Patient Cohorts** | | **Change in age profile for admissions between Pre-COVID and During COVID cohorts** | **Change in numbers of admissions for patients in During COVID cohorts** | **Pearson’s**  **Chi-squared Results (for change in age profile)** |
| --- | --- | --- | --- | --- | --- | --- | --- |
|  |  |  | **Pre-COVID average**  **(2017-19)** | **During COVID**  **(2020)** |  |  |  |
| **Female** | Age groups | 0-49 | 187 (11·5%) | 143 (10·4%) | -1.·1% | -23·5% | ***Χ^2^* (3, N = 6258) = 8.65.**  ***p* = .034** |
|  |  | 50-64 | 413 (25 ·3%) | 336 (24·5%) | -0·8% | -18·6% |  |
|  |  | 65-74 | 440 (27·0%) | 339 (24·7%) | -2·3% | -23·0% |  |
|  |  | 75+ | 589 (36·2%) | 554 (40·4%) | +4·2% | -5·9% |  |
|  | **Total for females** | | **1629 (100%)** | **1372 (100%)** | - | -15·8% |  |
| **Male** | Age groups | 0-49 | 167 (9·2%) | 151 (9·1%) | -0·1% | -9·6% | *Χ^2^* (3, N = 7125) = .321.  *p* = .956 |
|  |  | 50-64 | 434 (23 ·8%) | 403 (24·4%) | +0·6% | -7·1% |  |
|  |  | 65-74 | 533 (29·3%) | 474 (28 ·6%) | -0·7% | -11·1% |  |
|  |  | 75+ | 688 (37·8%) | 627 (37·9%) | +0·1% | -8·9% |  |
|  | **Total for males** | | **1823 (100%)** | **1655 (100%)** | - | -9·2% |  |
| **All genders** | Age groups | 0 to 49 | 354 (10·3%) | 294 (9·7%) | -0 ·6% | -16·9% | *Χ^2^* (3, N = 13383) = 4·75.  *p* = .191 |
|  |  | 50 to 64 | 847 (24·5%) | 739 (24·4%) | -0·1% | -12·8% |  |
|  |  | 65 to 74 | 974 (28·2%) | 813 (26·9%) | -1 ·3% | -16·5% |  |
|  |  | 75+ | 1277 (37·0%) | 1181 (39·0%) | +2·0% | -7·5% |  |
|  | **Total for all genders** | | **3452 (100%)** | **3027 (100%)** | - | -12·3% |  |

**Supplementary Material, Table 5:** Changes in admission rates between the two cohorts, by age and gender.
